# Therapeutic effect of percutaneous vertebroplasty and nonoperative treatment on osteoporotic vertebral compression fracture: A randomized controlled trial protocol

**DOI:** 10.1097/MD.0000000000020770

**Published:** 2020-07-02

**Authors:** Dongliang Wang, Dingwei Cang, Ya Wu, Siqing Wang

**Affiliations:** Department of Spinal Surgery, Yancheng NO.1 People's Hospital, Jiang Su, China.

**Keywords:** osteoporotic vertebral compression fractures, pain control, randomized controlled trial, study protocol, vertebroplasty

## Abstract

**Background:**

Osteoporosis and related complications have been increasing with the aging population. Osteoporotic vertebral compression fractures (OVCFs) are the most common among all osteoporotic fractures. The purpose of this study was performed to compare the efficiency and safety of vertebroplasty versus conservative treatment for acute OVCFs.

**Methods:**

The conduct of this study followed the Declaration of Helsinki principles and the reporting of this study adhered to the Consolidated Standards of Reporting Trials guidelines for randomized controlled trials. Written informed consent was obtained from every participant. Participants were randomly assigned (1:1) to receive either vertebroplasty or control group. The primary outcome was pain relief at 1 month and 1 year, measured with a Visual Analogue Scale score. The secondary outcomes were Roland-Morris Disability Questionnaire, short form score, European Quality of Life-5 Dimensions, and postoperative complications.

**Results:**

We hypothesize that vertebroplasty will provide a rapid decrease of pain and an early return to daily life activities compared with the control group.

**Trial registration:**

This study protocol was registered in Research Registry (researchregistry5624).

## 1 Introduction

Osteoporosis and related complications have been increasing with the aging population. Osteoporotic vertebral compression fractures (OVCFs) are the most common among all osteoporotic fractures. OVCFs result in an increase of the incidence of mortality and morbidity, causes back/lumbar pain as well as kyphosis deformity, and reduces the quality of life. Conservative treatment is the initial treatment approach in this type of fracture.^[1–3]^ It consists of bed rest, analgesic drug use, brace implementation, and physiotherapy. Vertebroplasty was first performed in a painful vertebral hemangioma case in 1984 in France. Its indication has been performed in trauma, osteoporotic fractures, malignant or benign spinal tumors, as well as vertebral osteonecrosis being modified in time. The most common area of usage is currently painful OVCFs. Vertebroplasty technic is a procedure involving the percutaneous injection of cement (polymethylmethacrylate) into the fracture line, with the help of a guide. The aim of this method is to increase the quality of life of the patient by reducing pain, eliminating the need to remain bedridden, and reducing the use of drugs.

Vertebroplasty, the injection of polymethylmethacrylate into the fractured vertebral body, is frequently used for symptomatic osteoporotic fractures, and is based on the premise that fracture stabilization can provide pain relief. It is a minimally invasive surgical technique first developed in the treatment of vertebral hemangiomas. Since its introduction, this minimally invasive technique has gained widespread recognition, effectively reducing pain both in the short and long term. The aim of this method is to increase the quality of life of the patient by reducing pain, eliminating the need to remain bedridden, and reducing the use of drugs.^[4,5]^ However, the effect of percutaneous cement augmentation are unclear, as are the benefits of vertebroplasty and its adverse procedure-related events and incidence of adjacent compression fractures. Two randomized studies with a sham control intervention have reported clinical outcomes 1 month and 6 months after percutaneous vertebroplasty in patients with osteoporotic vertebral fractures up to a year old. Results of both studies seem to show that vertebroplasty and sham treatment are equally effective.^[4,5]^

Recently, several studies have been published to compare vertebroplasty to conservative treatment of patients with OVCFs, but with different conclusions.^[6–11]^ We thus also designed a randomized controlled study to compare the efficiency and safety of vertebroplasty versus conservative treatment for acute OVCFs. We hypothesize that vertebroplasty will provide a rapid decrease of pain and an early return to daily life activities compared with the control group.

## 2 Material and method

### 2.1 Study design

This prospective, randomized, clinical trial was registered in Research Registry (researchregistry5624). The conduct of this study followed the Declaration of Helsinki principles and the reporting of this study adhered to the Consolidated Standards of Reporting Trials guidelines for randomized controlled trials. The Institutional Review Board of Yancheng NO.1 People's hospital also approved this study (IRB: YC1002037). Written informed consent was obtained from every participant.

### 2.2 Patients

Inclusion criteria were age ≥50 years, 1 to 3 vertebral compression fractures, T5 to L5 focal back pain at the level of fracture for up to 6 weeks, score of ≥5 on a VAS, ≥15% loss of vertebral height, and bone edema on magnetic resonance imaging. Exclusion criteria were inability to provide informed consent, chronic back pain requiring opiate use, substantial fracture retropulsion, acute infection, spinal malignancy, neurological complications, and >2 vertebral fractures.

### 2.3 Randomization and blinding

**Figure 1:**
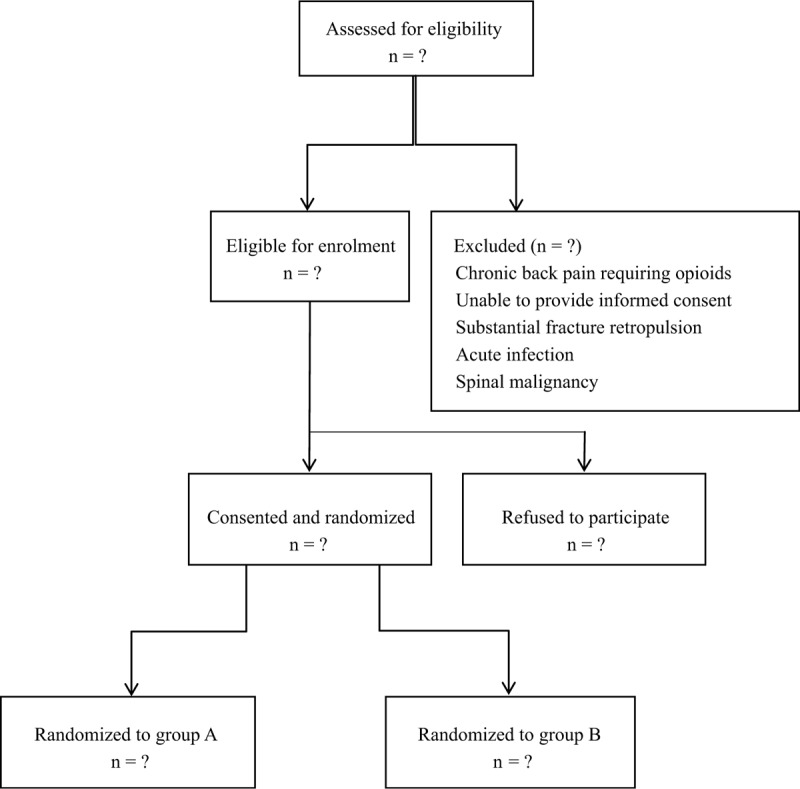
Flow diagram of the study.

Participants were randomly assigned (1:1) to receive either vertebroplasty or control group by the National Health and Medical Research Council Clinical Trials Centre automated telephone service, which provided random computer-generated numbers. The interventional radiologist called this system once the patient was in the procedure room, immediately before the procedure. Randomization was stratified according to age, vertebral height loss, trauma, steroid use, and intervention center. The participants, investigators (other than radiologists doing the procedure), and trial outcome assessors were masked to patient group assignments. To enhance masking, neither the radiologist nor staff at the treating centers had any other role in the trial (Fig. 1).

### 2.4 Intervention

The surgical approach was conducted in all cases under local anesthetic and intravenous sedation with the patient in the prone position. Local anesthesia of 2% prilocaine hydrochloride (6 mL) was applied from subcutaneous tissue as far as the pedicle entrance. In addition, 1.5 cm^3^ midazolam was administered intravenously for sedation. After location of the pedicle entrance of the fractured vertebra under fluoroscopy, an 11-gauge needle was entered from the pedicle. When the needle reached 2/3 anterior of the vertebral corpus on the lateral view, the cementing procedure began. The amount of cement was evaluated and defined under fluoroscopy. Monitoring during the procedure included electrocardiogram, oxygen saturation, and blood pressure. After the procedure, the patients were held in a prone position for 30 minutes and supine for further 90 minutes. Both groups were offered pain medication and physiotherapy if necessary until discharge. Additionally patients in the conservative group were offered brace treatment.

### 2.5 Outcome measures

The primary outcome was pain relief at 1 month and 1 year, measured with a Visual Analogue Scale (VAS) score ranging from 0 (no pain) to 10 (worst pain ever). We defined clinically significant pain relief as a decrease in VAS score from baseline of ≥3 points. Pain-free days were defined as days with a VAS score of ≤3.

The secondary outcomes were Roland-Morris Disability Questionnaire (RDQ), short form (SF-36) score, European Quality of Life-5 Dimensions (EQ-5D), and postoperative complications. The RDQ consisting of 24 questions about dysfunctions in daily activities experienced by patients with back pain. Scores range from 0 to 24, with higher numbers indicating worse physical functioning. SF-36 is a short-form health survey consisting of 36 questions covering 8 dimensions (physical function, social function, role physical, role emotional, mental health, vitality, bodily pain, and general health). From the 8 dimensions, 2 summary scores are formed: the Standardized physical component and the standardized mental component. European Quality of Life-5 Dimensions (EQ5D) is an instrument measuring health outcome and consists of 5 dimensions (mobility, self-care, usual activities, pain/discomfort, and anxiety/depression). Each dimension has 3 levels (no problems, moderate problems, and extreme problems). By combining the scores, 243 different unique health states are possible ranging from perfect health to worst possible health.

### 2.6 Sample size calculation

The sample size was determined for the primary endpoint and was calculated using PASS 2011 software (NCSS, LLC, Kaysville, UT). According to the results of our previous study, the postoperative VAS score for nausea was 2.16 in the control group. We anticipated a difference of 0.72 in the VAS score. With a power of 0.90 and significance level of 0.05, the required sample size was calculated as 70 in each arm. Considering possible exclusion, we decided to include 80 patients in each group.

### 2.7 Statistical analysis

All statistical analyses are performed using SPSS v. 24 (IBM Corp., Armonk, NY). Descriptive statistics of demographic and clinical characteristics are presented with mean standard deviation for continuous scale variables. The difference between normally distributed continuous scale variables is examined using Student *t* test, while non-normal variables are examined using Wilcoxon rank sum test. The association between categorical variables is examined using Pearson Chi-squared test or Fisher exact test. All analyses are performed in accordance with intention-to-treat principle.

## 3 Discussion

Osteoporosis and related complications have been increasing with the aging population. OVCFs are the most common among all osteoporotic fractures. OVCFs result in an increase of the incidence of mortality and morbidity, causes back/lumbar pain as well as kyphosis deformity, and reduces the quality of life. Conservative treatment is the initial treatment approach in this type of fracture. It consists of bed rest, analgesic drug use, brace implementation, and physiotherapy. Vertebroplasty was first performed in a painful vertebral hemangioma case in 1984 in France. Its indication has been performed in trauma, osteoporotic fractures, malignant or benign spinal tumors, as well as vertebral osteonecrosis being modified in time. The most common area of usage is currently painful OVCFs. Vertebroplasty technic is a procedure involving the percutaneous injection of cement (polymethylmethacrylate) into the fracture line, with the help of a guide.^[12–16]^ The aim of this method is to increase the quality of life of the patient by reducing pain, eliminating the need to remain bedridden, and reducing the use of drugs.

Three potential limitations to this study were identified. First, VAS pain was recorded by nursing pain assessment records in hospital and thus pain measurements are based on the frequency and accuracy of documentation in the medical record. The second limitation was the small sample size used in this study. Replication of this study on a multi-institutional level would provide less variation between experimental groups and simultaneously increase the reliability and generaliz-ability of the results. Finally, having the same surgeon for all procedures in this study was both an advantage and disadvantage. It is advantageous for consistency and internal validity; however, it may limit the reproducibility of this study.

## Author contributions

**Conceptualization:** Dongliang Wang.

**Data curation:** Dongliang Wang, Dingwei Cang.

**Formal analysis:** Dongliang Wang, Dingwei Cang.

**Funding acquisition:** Siqing Wang.

**Investigation:** Dongliang Wang, Dingwei Cang, Ya Wu.

**Methodology:** Dongliang Wang.

**Resources:** Siqing Wang.

**Software:** Dingwei Cang, Ya Wu.

**Supervision:** Siqing Wang.

**Validation:** Ya Wu.

**Visualization:** Ya Wu.

**Writing – original draft:** Dongliang Wang.

**Writing – review & editing:** Ya Wu, Siqing Wang.
